# Confined Internal Standard Core–Gap–Shell Nanoprobes for Ratiometric SERS Sensing of Urine pH

**DOI:** 10.3390/s26134205

**Published:** 2026-07-03

**Authors:** Xiao Wu, An Wang, Xiao Cai, Fan-Li Zhang, Bing Pei

**Affiliations:** 1 Zhejiang Province Key Laboratory of Optoelectronic Metrology and Precision Instruments, College of Optical and Electronic Technology, China Jiliang University, Hangzhou 310018, China; 18020224332@163.com (X.W.); zhangfl@cjlu.edu.cn (F.-L.Z.); 2 Jiangsu Province (Suqian) Hospital, Suqian 223800, China; donwenjun@126.com; 3 College of Plant Protection, Nanjing Agricultural University, Nanjing 210095, China

**Keywords:** ratiometric SERS sensing, urine pH detection, core–gap–shell nanostructure, confined internal standard

## Abstract

Urine pH is an important biomarker related to metabolic status and urinary system health, but reliable SERS quantification in real urine remains limited by matrix interference, heterogeneous hotspot distribution, and the narrow response range of single pH-responsive molecules. Here, we report a core–gap–shell Au@1,4-BDT@Au@4-MBA/MPY nanoprobe for ratiometric SERS detection of urine pH. 1,4-BDT was confined within the gap between the gold core and shell as an internal standard, while 4-MBA and 4-MPY were co-assembled on the outer gold shell to provide complementary protonation/deprotonation responses. The internal standard-corrected ratio I_1004_/I_1400_/I_731_ reduced signal fluctuation and enabled segmented linear fitting over pH = 1.0–7.0 and pH = 7.0–10.0, with coefficients of determination of 0.98806 and 0.99989, respectively. The sensing platform also maintained stable ratiometric responses under different interference conditions. In real urine samples from five volunteers, SERS-predicted pH values agreed well with commercial pH meter measurements, with relative accuracies of 98.71–101.9% and RSD values below 2.1%. This confined internal standard and dual-molecule ratiometric strategy provides a feasible approach for urine pH sensing in complex biofluid matrices.

## 1. Introduction

Surface-enhanced Raman scattering (SERS) has attracted considerable attention in biological fluid analysis, disease screening, and point-of-care testing because of its high sensitivity, molecular fingerprinting capability, rapid response, and low sample consumption [1,2,3,4]. Urine is an easily accessible and noninvasive biological fluid that reflects human metabolic status, renal function, and urinary system health [5,6]. Among its physicochemical parameters, pH is one of the most commonly used indicators with clinical relevance [7]. However, real urine samples contain a complex matrix composed of urea, uric acid, creatinine, inorganic salts, proteins, cell debris, and other components, which may induce nonspecific adsorption, background interference, and signal fluctuations. Therefore, achieving stable, accurate, and wide-range pH quantification in complex urine samples remains a key challenge for the practical application of SERS sensors [8].

Current SERS-based pH sensing generally relies on the protonation/deprotonation equilibrium of pH-responsive molecules under different acidic and alkaline conditions, and quantitative relationships are usually established based on changes in characteristic Raman peak intensities or peak positions [9,10]. Among these molecules, 4-mercaptobenzoic acid (4-MBA) and 4-mercaptopyridine (4-MPY) have been widely used for SERS pH detection because they can be stably immobilized on noble-metal surfaces through Au-S bonds and exhibit well-defined pH-responsive spectral features [11,12]. Nevertheless, the effective response range of a single pH-responsive molecule is usually limited by its intrinsic acid-base dissociation behavior, making it difficult to maintain continuous, sensitive, and stable responses over a broad pH range. In addition, SERS signals are highly dependent on local plasmonic hotspot distribution, molecular adsorption configuration, and substrate uniformity, resulting in fluctuations in absolute peak intensity and reduced reliability of quantitative models [13,14].

To improve the stability of SERS quantitative analysis, internal standard calibration strategies have received increasing attention [15,16,17,18,19,20,21]. In particular, core–shell plasmonic structures with subnanometer gaps can physically confine internal standard molecules within the gap between the core and shell [22,23,24,25]. On the one hand, the core–shell gap can generate strong electromagnetic coupling and significantly enhance the Raman signal of the internal standard molecules. On the other hand, the outer gold shell can isolate the internal standard molecules from the external complex environment, making them less susceptible to sample matrix effects, pH variations, and nonspecific adsorption [26,27,28,29]. This feature provides a stable reference signal for ratiometric SERS quantification. However, internal standard strategies mainly address signal fluctuation and do not directly broaden the effective detection range of pH-responsive molecules. If the outer surface relies on only one pH-responsive molecule, the sensing performance remains constrained by the intrinsic pKa value and response window of that molecule [30,31].

Based on this consideration, we propose a “confined internal standard calibration-dual-molecule ratiometric response” strategy for SERS pH sensing. Unlike conventional SERS pH sensing methods that rely on a single responsive molecule or external peak ratios, this work spatially separates the stable reference signal from the pH-responsive signal. Specifically, 1,4-benzenedithiol (1,4-BDT) is confined within the subnanometer gap between the gold core and gold shell, serving as an internal reference signal that is not directly exposed to the external solution environment. Meanwhile, 4-MBA and 4-MPY are co-assembled on the outer gold shell surface, providing pH responses based on carboxyl and pyridine groups, respectively. Because these two molecules exhibit complementary protonation/deprotonation behaviors in different acidic and alkaline ranges, this structure simultaneously addresses signal fluctuation in SERS quantification and the limited response range of single-molecule probes, enabling the construction of a wide-range and fluctuation-resistant ratiometric pH calibration model.

As a proof of concept for complex biological sample analysis, the proposed sensing platform was further applied to pH determination in real human urine samples. Different from previous urine SERS studies that mainly focused on substrate flexibility, sample pretreatment, or multianalyte detection, this work emphasizes the stabilization of pH quantification in urine through a confined internal standardized ratiometric nanoprobe design. By comparing the SERS-predicted pH values with those measured using a commercial pH meter, we evaluated the accuracy and anti-interference capability of the platform in complex urine matrices. This work not only provides a new SERS analytical approach for rapid and noninvasive urine pH detection but also offers a useful structural design strategy for developing ratiometric SERS sensors with improved quantitative stability in complex biological fluids.

## 2. Materials and Methods

### 2.1. Materials

Chloroauric acid (HAuCl_4_·4H_2_O), sodium chloride (NaCl, AR), and calcium chloride (CaCl_2_) were purchased from Sinopharm Chemical Reagent Co., Ltd. (Shanghai, China). Cetyltrimethylammonium chloride (CTAC, 99%), 1,4-benzenedithiol (1,4-BDT, 98%), 4-mercaptobenzoic acid (4-MBA), 4-mercaptopyridine (4-MPY), ascorbic acid (AA, 99%), and dichloromethane were purchased from Aladdin (Shanghai, China). Absolute ethanol was purchased from Gaojing Chemical Co., Ltd. (Hangzhou, China). Disodium hydrogen phosphate (Na_2_HPO_4_, 99%), sodium formate (HCOONa, AR), barium chloride dihydrate (BaCl_2_·2H_2_O, AR), potassium chloride (KCl, AR), magnesium sulfate (MgSO_4_, AR), and cyclohexane were purchased from Rohn Reagent (Shanghai, China). Ultrapure water (>18.0 MΩ·cm) was used in all experiments.

### 2.2. Instruments and Characterization

The UV-vis absorption spectra of the nanoparticles were recorded using a UV-vis spectrophotometer (UV-MINI-1280, Shimadzu, Kyoto, Japan). The morphology of the nanoparticles and self-assembled gold films was characterized by scanning electron microscopy (SEM, S4800, Hitachi, Tokyo, Japan) and transmission electron microscopy (TEM, Hitachi, Japan). Scanning transmission electron microscopy (STEM) and energy-dispersive X-ray spectroscopy (EDS) were used to analyze the elemental distribution. SERS spectra were collected using a micro-Raman spectrometer (VKR-S, SHINs, Xiamen, China) equipped with a 785 nm excitation laser and a 50× objective.

### 2.3. Preparation of Au@1,4-BDT@Au@4-MBA/MPY

The 40 nm gold nanospheres (AuNSs) were prepared using a modified seed-mediated growth method. First, 50 μL of HAuCl_4_ (0.05 M) was added to 5 mL of CTAC (0.1 M) solution, followed by the rapid injection of 200 μL of ice-cold NaBH_4_ (0.02 M) under vigorous stirring. After 3 min, the obtained seed solution was diluted 10-fold with 100 mM CTAC. Subsequently, 900 μL of the diluted seed solution and 40 μL of AA (0.1 M) were added to 10 mL of CTAC (25 mM) solution. Then, 50 μL of HAuCl_4_ (0.05 M) was added, and the mixture was left undisturbed for 10 min to obtain approximately 10 nm gold seeds. Next, 900 μL of the gold seed solution, 200 mL of CTAC (25 mM), and 800 μL of AA (0.1 M) were mixed and stirred at room temperature for 1 h, followed by the slow addition of 1 mL of HAuCl_4_ (50 mM) within 10 min. Under vigorous stirring, 200 μL of NaClO and 50 μL of HAuCl_4_ (50 mM) were sequentially added, and the mixture was stirred for another 45 min to obtain 40 nm AuNS solution. All reactions were performed at room temperature.

For the growth of Au@BDT@Au core–shell nanoparticles and subsequent functionalization with 4-MBA/4-MPY, 100 mL of the 40 nm AuNS solution was centrifuged twice (7500 rpm, 15 min) to reduce the concentration of surfactant. The collected nanoparticles were then reacted with 1 mL of 1,4-BDT (1 mM) for 0.5 h. Excess molecules were removed by three cycles of centrifugation and washing, and the nanoparticles were redispersed in 0.1 mM CTAC solution. Subsequently, 1 mL of HAuCl_4_ (5 mM) and 500 μL of AA (0.1 M) were added to 10 mL of CTAC (0.1 M) solution, followed by the injection of 1 mL of Au@BDT seeds under stirring for 30 min to achieve gold shell growth. Finally, 100 μL of 4-MBA (1 mM) and 100 μL of 4-MPY (1 mM) were simultaneously added and reacted for 1 h to functionalize the outer gold shell. The resulting Au@1,4-BDT@Au@4-MBA/MPY nanoparticles were washed three times by centrifugation (3000 rpm, 5 min). Liquid-phase self-assembly was then performed using dichloromethane and cyclohexane, and the formed gold film was carefully transferred onto a silicon substrate using tweezers.

### 2.4. SERS Measurement and pH Sensing Procedure

To construct the pH calibration curve, the silicon substrate loaded with the self-assembled gold film was placed in a small Petri dish. Then, 3 mL of standard buffer solution with a specific pH value was slowly added until the gold film was completely immersed. SERS spectra were collected directly from the immersed substrate. The solutions for pH = 1.0, 2.0, 3.0, and 4.0 were prepared by titrating a commercial pH = 4.0 sodium hydrogen phthalate buffer with HCl and NaOH. The solutions for pH = 5.0, 6.0, and 7.0 were obtained by titrating a commercial pH = 6.86 mixed phosphate buffer with HCl and NaOH. Finally, the solutions for pH = 8.0, 9.0, and 10.0 were prepared by titrating a commercial pH = 9.18 borate buffer with HCl and NaOH.

For real urine testing, midstream morning urine samples were collected from five healthy volunteers. After simple centrifugation to remove cell debris, precipitates, and suspended particles, 3 mL of the supernatant was used to immerse the SERS chip for spectral acquisition. All urine samples were collected with informed consent from the volunteers. The laser power was set to 75 mW, the integration time was 1 s, and one accumulation was used for each spectrum.

## 3. Results and Discussion

**Figure 1:**
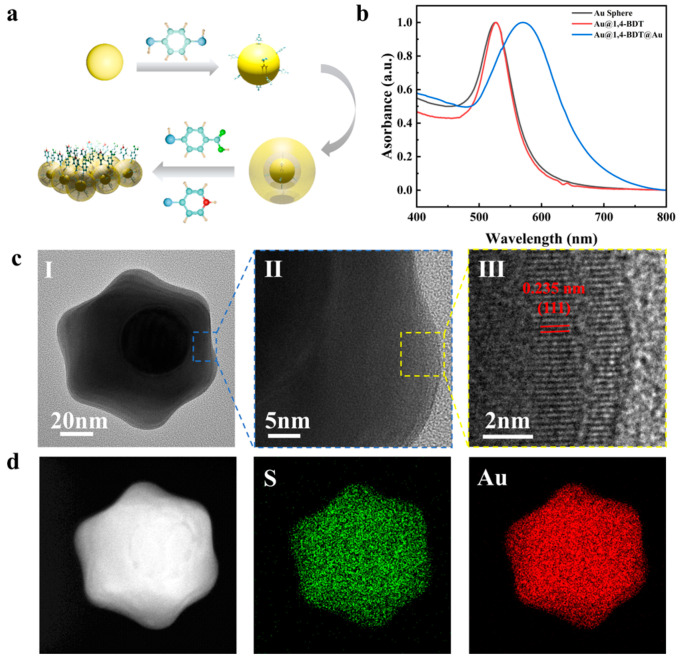
Design, synthesis, and structural characterization of the Au@1,4-BDT@Au@4-MBA/MPY core–gap–shell nanoprobes. (**a**) Schematic illustration of the nanoprobe preparation process. (**b**) UV-vis absorption spectra of nanoparticles at different assembly stages. (**c**) TEM images of Au@1,4-BDT@Au nanoparticles. (I) Single particle; (II) Local enlargement of the blue box area; (III) High-resolution lattice-fringe image of yellow box area. (**d**) EDS elemental analysis of Au@1,4-BDT@Au@4-MBA/MPY nanoprobes.

To integrate internal standard calibration with pH-responsive sensing, Au@1,4-BDT@Au@4-MBA/MPY core–gap–shell composite nanoprobes were constructed, as illustrated in Figure 1a. 1,4-BDT was first modified onto the surface of gold nanocores through Au-S interactions and was subsequently confined between the gold core and the outer gold shell through in situ shell growth, forming an internal standard signal that was not directly exposed to the external solution environment. 4-MBA and 4-MPY were further co-assembled on the outer gold shell surface as pH-responsive molecules for directly sensing changes in solution pH. In this way, the internal reference signal and the external responsive signal were spatially separated, providing the structural basis for subsequent ratiometric pH detection.

The UV-vis absorption spectra of nanoparticles at different assembly stages reflected the gradual evolution of the plasmonic structure (Figure 1b). Bare gold nanoparticles exhibited a typical localized surface plasmon resonance peak at approximately 526.5 nm. After modification with 1,4-BDT, a slight red shift was observed, indicating a change in the local dielectric environment around the gold core surface [32]. After further growth of the gold shell, the absorption peak became broadened and further red-shifted, which is consistent with the formation of the core–shell structure and the enhanced plasmonic coupling associated with the core–gap–shell geometry [33]. These optical changes support the successful construction of the core–gap–shell structure and provide the basis for SERS enhancement of the confined internal standard molecules.

Transmission electron microscopy further confirmed the morphology and structural features of the nanoprobes (Figure 1c). The Au@1,4-BDT@Au nanoparticles exhibited a quasi-spherical morphology with clear core–shell contrast, indicating successful coating of the outer gold shell on the gold core. High-resolution TEM images showed lattice fringes with a spacing of approximately 0.235 nm, corresponding to the (111) plane of face-centered cubic gold, suggesting good crystallinity of the outer gold shell. The continuous gold shell helps confine 1,4-BDT molecules within the core–shell gap and provides the gold surface required for subsequent 4-MBA/4-MPY co-assembly.

In addition, EDS elemental analysis showed the coexistence of Au and S elements in the nanoparticles (Figure 1d), indicating that sulfur-containing molecules had been successfully introduced into the nanostructure. Since 1,4-BDT, 4-MBA, and 4-MPY can all bind to gold surfaces through Au-S interactions, the presence of sulfur supports the successful molecular modification from a compositional perspective. Taken together, the UV-vis absorption spectra, TEM morphology, and EDS elemental analysis confirm the successful construction of the Au@1,4-BDT@Au@4-MBA/MPY composite nanoprobes. The resulting structure contains both confined internal standard signals in the gap region and dual-molecule pH-responsive sites on the outer surface, laying the foundation for constructing a fluctuation-resistant and wide-range ratiometric SERS pH sensing model.

**Figure 2:**
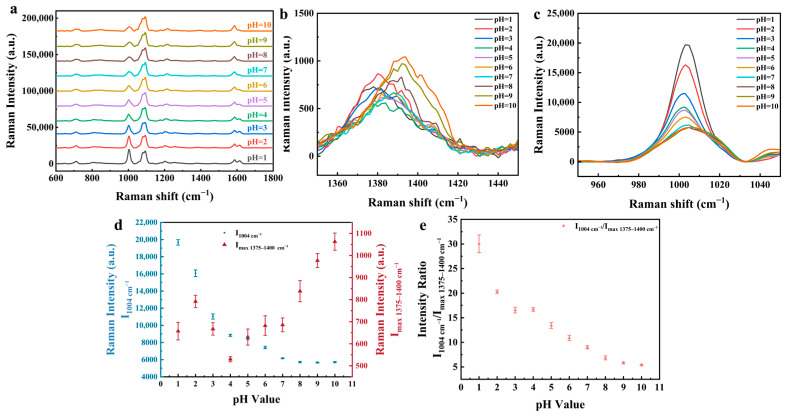
Verification of the pH-responsive behavior of 4-MBA/MPY dual-responsive molecules on a gold-film substrate. (**a**) SERS spectra of the dual-molecule-modified gold film under different pH conditions. (**b**) Magnified spectra of the symmetric stretching vibration peak of the 4-MBA carboxylate group. (**c**) Magnified spectra of the pyridine ring breathing vibration peak of 4-MPY. (**d**) Intensity variations of the 1400 cm^−1^ and 1004 cm^−1^ characteristic peaks as a function of pH. (**e**) Variation of the dual-peak ratio I_1004_/I_1400_ with pH.

Before constructing the core–gap–shell composite probes with an internal standard, it was necessary to verify the pH-responsive behavior of 4-MBA and 4-MPY dual-responsive molecules on the same plasmonic interface. Therefore, a two-dimensional gold film was used as a simplified SERS substrate. 4-MBA and 4-MPY were co-modified on the surface, and SERS spectra were collected in buffer solutions with different pH values. As shown in Figure 2a, the characteristic peaks of both 4-MBA and 4-MPY appeared on the gold film, indicating that the two responsive molecules could coexist on the same gold surface and generate distinguishable SERS signals.

Further analysis of the two key response peaks revealed that 4-MBA and 4-MPY exhibited different pH-dependent behaviors. As shown in Figure 2b, the peak at approximately 1400 cm^−1^ is mainly assigned to the symmetric stretching vibration of the carboxylate group in 4-MBA [34]. With increasing solution pH, the carboxyl group gradually underwent deprotonation, leading to an intensification of the signal associated with the symmetric stretching vibration of the carboxylate ion. Concurrently, this deprotonation promotes electron delocalization from the aromatic ring to the carboxylate, which leads to an increase in the vibrational frequency and causes a blue shift in this peak. This indicates that 4-MBA shows a pronounced response in the neutral-to-alkaline range. In contrast, the peak at approximately 1004 cm^−1^ in Figure 2c is associated with the pyridine ring breathing vibration of 4-MPY [35]. Its intensity is mainly regulated by the protonation/deprotonation state of the pyridine nitrogen atom and exhibits a pH response behavior distinct from that of 4-MBA. Specifically, under acidic conditions, the nitrogen atom on the pyridine ring binds a proton to form a pyridinium cation, resulting in a strong signal and a highly sensitive response. As the pH increases, it gradually loses a proton to return to a neutral pyridine molecule, causing the intensity of the ring breathing peak to progressively attenuate.

The pH-dependent changes of the 1400 cm^−1^ and 1004 cm^−1^ peaks were further analyzed to evaluate their suitability for ratiometric readout (Figure 2d). Based on their complementary response characteristics, the dual-peak ratio I_1004_/I_1400_ was constructed (Figure 2e). The continuous decrease of I_1004_/I_1400_ with increasing pH confirms that 4-MBA and 4-MPY can provide coordinated pH responses. While this complementary dual-molecule strategy successfully broadens the dynamic detection range beyond the intrinsic limits of a single probe, the uncalibrated peak ratio remains susceptible to absolute signal fluctuations caused by heterogeneous hotspot distributions and complex matrices. To address this, an independent internal standard was subsequently introduced to construct a three-peak ratiometric system, integrating the wide-range responsive advantages of the dual probes with the quantitative stability provided by internal calibration.

**Figure 3:**
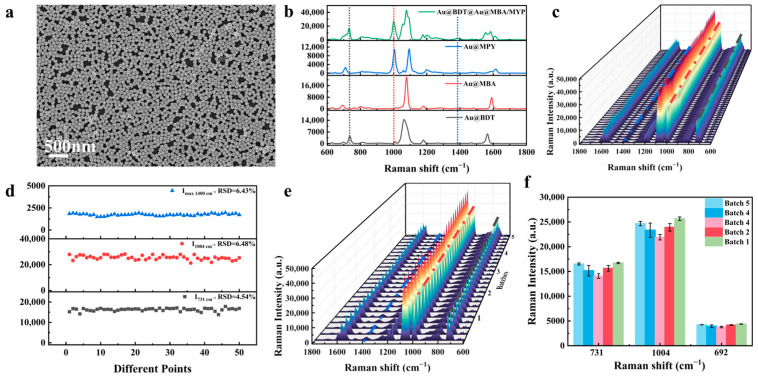
Morphology and signal reproducibility characterization of the Au@1,4-BDT@Au@4-MBA/MPY composite SERS substrate. (**a**) SEM image of the self-assembled gold-film substrate. (**b**) SERS spectra of single-component probes and the final composite probe. (**c**) SERS spectra collected from 50 randomly selected points on a single substrate. (**d**) Point-to-point intensity distribution of the 1004 cm^−1^, 1400 cm^−1^, and 731 cm^−1^ characteristic peaks. (**e**) SERS spectra collected from random points on five independent gold-film substrates from the same batch. (**f**) Statistical analysis of characteristic peak intensities from different substrates.

The reliability of SERS-based quantitative detection depends not only on the pH sensitivity of the responsive molecules but also on the uniformity of hotspot distribution and signal reproducibility of the substrate. Therefore, before evaluating the pH response, the morphology and SERS signal consistency of the Au@1,4-BDT@Au@4-MBA/MPY composite sensing substrate were first examined. As shown in Figure 3a, the self-assembled Au@1,4-BDT@Au nanoparticles formed a relatively dense and continuous film on the silicon substrate. The close spacing between adjacent nanoparticles favors the formation of localized plasmonic hotspots in the interparticle gaps, providing the structural basis for stable SERS enhancement.

The successful integration of the internal standard and the two pH-responsive molecules was verified by comparing the SERS spectra of the single-component probes and the final composite probe (Figure 3b). The final probe simultaneously retained the characteristic peaks of 1,4-BDT, 4-MBA, and 4-MPY. Specifically, the peak at 731 cm^−1^ originates from 1,4-BDT confined within the core–shell gap and serves as an internal reference signal [36,37]; the peak near 1400 cm^−1^ is assigned to the carboxylate-related vibration of 4-MBA [38,39]; and the peak at 1004 cm^−1^ corresponds to the pyridine ring breathing vibration of 4-MPY [40,41]. The coexistence of these three peaks provides the spectral basis for subsequent three-peak ratiometric analysis.

To further evaluate the signal uniformity within a single substrate, SERS spectra were collected from 50 randomly selected positions on the same gold-film substrate. As shown in Figure 3c, the spectral profiles obtained from different points were highly consistent, and the three key characteristic peaks were all clearly observed. The corresponding peak-intensity statistics are shown in Figure 3d. The relative standard deviations of the 1004 cm^−1^, 1400 cm^−1^, and 731 cm^−1^ peaks were 6.43%, 6.48%, and 4.54%, respectively, indicating good microscale signal uniformity of the self-assembled substrate. Notably, the smaller fluctuation of the 731 cm^−1^ internal standard peak suggests that the confined internal reference signal remains relatively stable at different detection positions, which helps reduce quantitative errors caused by local hotspot variations.

Inter-substrate reproducibility was further evaluated using five independent gold-film substrates prepared from the same batch. For each substrate, SERS spectra were collected from five randomly selected positions. The spectra obtained from different substrates showed consistent profiles (Figure 3e), and the corresponding intensity statistics indicated acceptable peak-intensity variations among substrates (Figure 3f). Overall, the self-assembled composite substrate showed acceptable point-to-point uniformity and substrate-to-substrate reproducibility, supporting the following ratiometric pH calibration.

**Figure 4:**
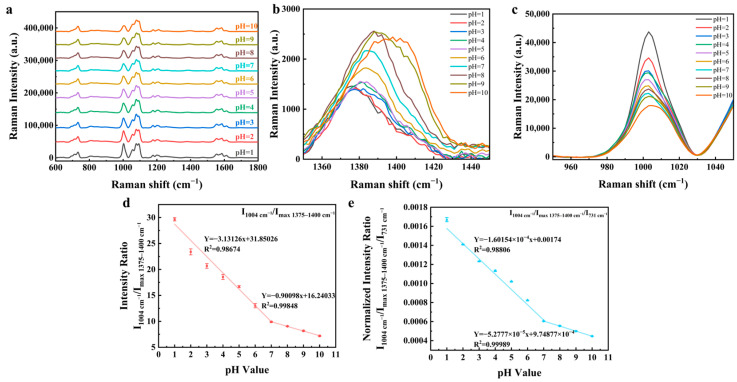
pH-responsive performance and ratiometric calibration model of the Au@1,4-BDT@Au@4-MBA/MPY composite probe. (**a**) SERS spectra of the composite probe under different pH conditions. (**b**) Magnified spectra of the symmetric stretching vibrations of the carboxylate group at 1400 cm^−1^. (**c**) Magnified spectra of the pyridine ring breathing vibration at 1004 cm^−1^. (**d**) Fitting relationship between the dual-peak ratio I_1004_/I_1400_ and pH. (**e**) Fitting relationship between pH and the three-peak corrected ratio I_1004_/I_1400_/I_731_ after calibration with the 731 cm^−1^ internal standard peak.

After confirming substrate uniformity and reproducibility, the pH-responsive performance of the Au@1,4-BDT@Au@4-MBA/MPY composite nanoprobe was evaluated in buffer solutions with different pH values. The SERS spectra exhibited regular pH-dependent changes (Figure 4a). The characteristic peaks of the outer 4-MBA and 4-MPY molecules changed significantly with pH, whereas the 1,4-BDT peak at 731 cm^−1^ remained relatively stable throughout the testing range. This result confirms that the gap-confined 1,4-BDT does not directly participate in the external acid-base response and can be used as an internal reference for intensity correction.

Further analysis of the two key response peaks revealed complementary pH-responsive behaviors of 4-MBA and 4-MPY. As shown in Figure 4b, the 4-MBA peak of the symmetric stretching vibration of the carboxylate group near 1400 cm^−1^ gradually increased with increasing pH, indicating enhanced deprotonation of the carboxyl group and a stronger carboxylate vibration signal. In contrast, the 4-MPY pyridine ring breathing vibration peak at 1004 cm^−1^ gradually decreased with increasing pH (Figure 4c), mainly owing to the transition of the pyridine nitrogen atom from a protonated state to a deprotonated state. These trends are consistent with the results of the preliminary dual-molecule experiment without an internal standard, confirming that 4-MBA/4-MPY retained effective pH responsiveness after being incorporated into the core–gap–shell probe.

Based on these two response peaks, the dual-peak intensity ratio I_1004_/I_1400_ was first constructed without internal standard correction. This ratio was used to describe the relative pH-responsive relationship between the two responsive molecules. As shown in Figure 4d, I_1004_/I_1400_ decreased markedly with increasing pH and exhibited a segmented response. In the pH = 1.0–7.0 range, the protonated-to-deprotonated transition of 4-MPY was more pronounced, and the decrease in the 1004 cm^−1^ peak intensity contributed more strongly to the decline of the ratio. In the pH = 7.0–10.0 range, the response of 4-MPY gradually became less pronounced, while the increase in the 1400 cm^−1^ peak intensity caused by further deprotonation of the 4-MBA carboxyl group became the dominant factor affecting the ratio. Therefore, the dual-responsive molecule system enables a relay-like response from acidic to weakly alkaline conditions, thereby broadening the effective detection range of a single pH probe.

To further reduce the influence of local hotspot variation, laser power fluctuation, and microscale substrate heterogeneity on quantitative results, the 1,4-BDT internal standard peak at 731 cm^−1^ was introduced for correction, and the three-peak corrected ratio I_1004_/I_1400_/I_731_ was constructed. This ratio preserves the complementary response information from the 4-MBA/4-MPY dual-molecule system while incorporating the confined internal standard signal to compensate for intensity fluctuations during SERS measurement. As shown in Figure 4e, after internal standard correction, the dispersion of the calibration curve was significantly reduced. The linear fitting coefficients in the pH = 1.0–7.0 and pH = 7.0–10.0 ranges increased to 0.98806 and 0.99989, respectively. The improvement after introducing I_731_ highlights the role of the confined 1,4-BDT signal in compensating for intensity fluctuations, while the 4-MBA/4-MPY pair maintains the broad pH response.

Complex biological fluids typically contain inorganic ions and small-molecule metabolites, which may affect SERS detection by changing the ionic strength of the solution, inducing competitive adsorption, or introducing background signals [42]. Therefore, before testing real urine samples, it was necessary to evaluate the response stability of the Au@1,4-BDT@Au@4-MBA/MPY composite probe against common interfering species. For this purpose, three representative pH conditions, namely pH = 4.0, pH = 7.0, and pH = 9.18, were selected to represent acidic, neutral, and weakly alkaline environments, respectively. Interfering species including NaCl, KCl, CaCl_2_, BaCl_2_, HCOONa, Na_2_HPO_4_, and MgSO_4_ were added to evaluate the SERS spectral changes of the composite probe under different matrix conditions.

**Figure 5:**
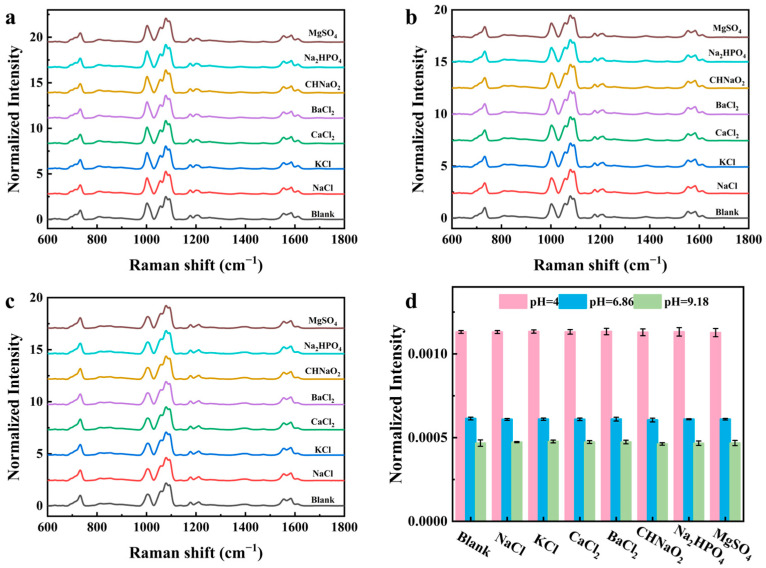
Anti-interference performance evaluation of the Au@1,4-BDT@Au@4-MBA/MPY composite probe. (**a**) SERS spectra in the presence of different interfering species at pH = 4.0. (**b**) SERS spectra in the presence of different interfering species at pH = 7.0. (**c**) SERS spectra in the presence of different interfering species at pH = 9.18. (**d**) Statistical results of I_1004_/I_1400_/I_731_ under different pH and interference conditions.

Under the same pH condition, the overall SERS spectral profiles of the composite probe remained essentially unchanged after the introduction of different interfering species (Figure 5a–c). The pyridine ring breathing vibration peak of 4-MPY at 1004 cm^−1^, the carboxylate-related peak of 4-MBA near 1400 cm^−1^, and the internal standard peak of 1,4-BDT at 731 cm^−1^ were all clearly distinguishable. No obvious new impurity peaks or significant peak shifts were observed, indicating that the selected interfering species did not significantly disrupt the pH-responsive behavior of the outer responsive molecules or affect the confined internal standard signal.

The anti-interference capability was further quantified using the I_1004_/I_1400_/I_731_ values obtained under different interference conditions (Figure 5d). Under the same pH condition, different interfering species caused only small variations in I_1004_/I_1400_/I_731_, whereas clear distinctions were maintained among different pH conditions. This result indicates that the ratiometric response of the composite probe is mainly governed by the acid-base state of the system rather than by individual salt ions or small-molecule interferents. Compared with detection methods based directly on single-peak intensity, the internal standard-corrected ratiometric signal can more effectively reduce the influence of local substrate variation, measurement fluctuation, and matrix background on quantitative results.

This anti-interference capability originates from the synergistic effect of the composite structure and the response mechanism. On the one hand, the outer 4-MBA and 4-MPY molecules respond to pH variations through protonation/deprotonation equilibria and exhibit relatively weak direct responses to common inorganic salts and small-molecule backgrounds. On the other hand, 1,4-BDT is confined within the gold core–shell gap, and its signal is not directly exposed to the external solution environment, enabling it to serve as a relatively stable internal reference. Thus, the ratio response remained mainly governed by pH rather than by the tested salts or small-molecule interferents, supporting subsequent measurements in real urine samples.

In addition to anti-interference capability, response reversibility is another important indicator for evaluating the practical applicability of pH sensors. An ideal pH-responsive probe should be able to switch repeatedly between acidic and alkaline environments while maintaining stable and reproducible spectral responses. Therefore, the Au@1,4-BDT@Au@4-MBA/MPY composite substrate was alternately immersed in pH = 1.0 and pH = 10.0 buffer solutions for 10 consecutive acid-base switching cycles to evaluate its response reversibility and signal stability.

**Figure 6:**
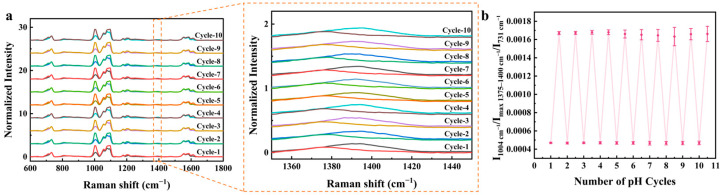
Acid-base cycling response reversibility of the Au@1,4-BDT@Au@4-MBA/MPY composite probe. (**a**) SERS spectra during alternating switching between pH = 1.0 and pH = 10.0. (**b**) Variation of I_1004_/I_1400_/I_731_ during 10 acid-base switching cycles.

During repeated acid-base switching, the SERS spectra of the composite probe showed regular transitions between two response states (Figure 6a). Under acidic conditions, the 4-MPY-related peak at 1004 cm^−1^ was relatively enhanced; after switching to alkaline conditions, the 4-MBA-related peak at 1400 cm^−1^ increased. This trend agrees with the pH calibration results, indicating that the outer 4-MBA and 4-MPY molecules retain reversible protonation/deprotonation responses. Meanwhile, the 1,4-BDT internal standard peak at 731 cm^−1^ remained clearly distinguishable without obvious peak shift or intensity attenuation, suggesting good short-term stability of the confined internal reference.

To further quantify the response reproducibility during cycling, the I_1004_/I_1400_/I_731_ value was extracted from each cycle, as shown in Figure 6b. The I_1004_/I_1400_/I_731_ value exhibited periodic variation between the pH = 1.0 and pH = 10.0 states, and the signal levels at high and low pH remained clearly distinguishable after 10 cycles. This result indicates that the ratiometric response of the composite probe is not an irreversible one-time change but originates from the reversible acid-base equilibrium of the outer responsive molecules. At the same time, the stable internal standard peak helps correct possible variations in detection position and local signal intensity during the cycling process.

Overall, these results demonstrate that the Au@1,4-BDT@Au@4-MBA/MPY composite sensing interface exhibits good acid-base response reversibility and short-term cycling stability. The reversible response mainly arises from the protonation/deprotonation equilibria of the 4-MBA carboxyl group and the 4-MPY pyridine group, while the 1,4-BDT confined within the core–gap–shell structure provides a stable internal reference during cycling. This performance further supports its use for repeatable pH detection in complex biological fluid samples.

**Figure 7:**
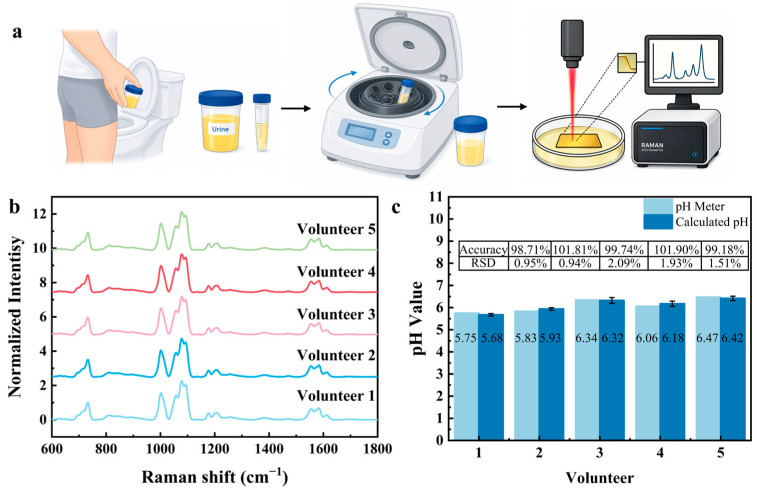
Validation of the Au@1,4-BDT@Au@4-MBA/MPY composite sensing platform for pH detection in real urine samples. (**a**) Schematic workflow of urine collection, simple centrifugation, SERS chip immersion, and spectral acquisition. (**b**) SERS spectra of urine samples from five volunteers. (**c**) Comparison between SERS-predicted pH values and those measured using a commercial pH meter.

After completing pH calibration in standard buffer solutions, anti-interference testing, and acid-base cycling validation, the applicability of the Au@1,4-BDT@Au@4-MBA/MPY composite sensing platform was further evaluated in real urine samples. Urine contains urea, uric acid, creatinine, inorganic salts, and other metabolites, making its matrix composition much more complex than that of standard buffer solutions. Therefore, it is suitable for evaluating the detection stability of this ratiometric SERS method in real biological samples. Midstream morning urine samples were collected from five healthy volunteers, simply centrifuged to remove suspended particles and precipitates, and then directly used for SERS detection. The real-sample testing workflow, including volunteer urine collection, simple centrifugation, immersion of the gold-film SERS chip in the centrifuged urine, and SERS spectral acquisition, is summarized in Figure 7a.

Clear and distinguishable SERS spectra were obtained from all five real urine samples (Figure 7b). The characteristic peaks of 4-MPY, 4-MBA, and 1,4-BDT were stably observed, indicating that the composite probe maintained effective SERS activity in the urine matrix. The outer 4-MBA/4-MPY molecules provided pH-related response signals, while the gap-confined 1,4-BDT served as the internal reference. These spectral features provide the basis for subsequent quantitative analysis.

Subsequently, the I_1004_/I_1400_/I_731_ values of the urine samples were extracted and substituted into the segmented calibration model established above to calculate the corresponding SERS-predicted pH values. To evaluate the accuracy of this method, the same urine samples were measured in parallel using a commercial pH meter, and the results obtained by the two methods were compared. As shown in Figure 7c, the SERS-predicted pH values agreed well with the pH meter measurements, indicating that the ratiometric SERS sensing platform can provide reliable pH estimation in real urine samples. According to the statistical results, the relative accuracy of the SERS method compared with the pH meter was 98.71–101.9%, with RSD values below 2.1%, demonstrating good detection repeatability and method agreement in small-sample real urine testing.

The real-sample results therefore support the feasibility of the “confined internal standard calibration-dual-molecule ratiometric response” strategy for complex biological fluid analysis. On the one hand, the dual-responsive design of 4-MBA and 4-MPY broadens the pH response range and enables coverage of the common urine pH window. On the other hand, the 1,4-BDT internal standard signal corrects intensity fluctuations during SERS measurement, thereby improving quantitative stability in real-sample analysis. Therefore, this composite SERS chip can serve as a candidate method for rapid urine pH detection and provides a useful reference for the design of ratiometric SERS sensors for noninvasive biological fluid analysis.

Meanwhile, a comparison of our method with recently reported pH detection strategies is summarized in Table 1. The proposed SERS platform effectively circumvents interferences from the natural color of biological fluids and matrix autofluorescence. Furthermore, it exhibits great potential for the simultaneous multiplexed detection of various biomarkers, such as urea, uric acid, and creatinine.

## 4. Conclusions

In summary, this work developed a ratiometric SERS sensing platform based on Au@1,4-BDT@Au@4-MBA/MPY core–gap–shell nanoprobes for quantitative pH detection in complex biological fluids. In this structure, 1,4-BDT internal standard molecules were confined within the subnanometer gap between the gold core and gold shell, allowing them to provide a relatively stable internal reference signal under external acid-base variations and complex matrix conditions. Meanwhile, the co-assembled 4-MBA and 4-MPY molecules on the outer gold shell provided pH responses associated with carboxyl and pyridine groups, respectively, forming a complementary dual-molecule response mode.

Experimental results demonstrated that the composite SERS substrate exhibited good signal uniformity and reproducibility, with relative standard deviations below 7% for the three characteristic peaks at 1004 cm^−1^, 1400 cm^−1^, and 731 cm^−1^. In the pH calibration experiments, the internal standard-corrected ratiometric model achieved fitting coefficients of 0.98806 and 0.99989 in the pH = 1.0–7.0 and pH = 7.0–10.0 ranges, respectively, indicating that confined internal standard calibration can effectively reduce the influence of SERS signal fluctuation on quantitative results. In addition, the sensing platform maintained stable ratiometric responses in the presence of common inorganic salts and small-molecule interferents, and exhibited good response reversibility during acid-base cycling between pH = 1.0 and pH = 10.0.

Furthermore, the method was applied to pH detection in real urine samples from five volunteers. The SERS-predicted results agreed well with those obtained using a commercial pH meter, with relative accuracies of 98.71–101.9% and RSD values below 2.1%. These results indicate that the “confined internal standard calibration-dual-molecule ratiometric response” strategy improves the quantitative stability of SERS sensors in complex urine matrices and provides a feasible analytical method for rapid and noninvasive urine pH detection. This design concept may also provide useful guidance for developing ratiometric SERS sensing platforms for complex biological fluid analysis. Beyond urine, it holds significant potential for the non-invasive monitoring of other biofluids, such as sweat and saliva. Furthermore, its robust anti-interference capability makes it highly suitable for environmental monitoring applications, including the pH determination of lake water and industrial wastewater.

## Figures and Tables

**Table 1:** Comparison of Different Methods.

Author	Method	pH Range	R^2^
Q.-L., Chen et al. [43]	Fluorescence	4.12–7.05	0.9914
X.-R., Zhang et al. [44]	Fluorescence	3.2–6.8	0.9975
F. Lorestani et al. [45]	Electrochemistry	4–8	0.97
M. R. Adib et al. [46]	Electrochemistry	5–8	0.997
L.-J., Zhou et al. [47]	Colorimetry	4–10	/
W.-A., Zhang et al. [48]	Colorimetry	3–7	/
This Work	SERS	1–7 7–10	0.98806 0.99989

## Data Availability

The original contributions presented in this study are included in the article. Further inquiries can be directed to the corresponding authors.
